# Fidgetin-like 2 negatively regulates axonal growth and can be targeted to promote functional nerve regeneration

**DOI:** 10.1172/jci.insight.138484

**Published:** 2021-05-10

**Authors:** Lisa Baker, Moses Tar, Adam H. Kramer, Guillermo A. Villegas, Rabab A. Charafeddine, Olga Vafaeva, Parimala Nacharaju, Joel Friedman, Kelvin P. Davies, David J. Sharp

**Affiliations:** 1 Department of Physiology and Biophysics,; 2 Department of Urology,; 3 Dominick P. Purpura Department of Neuroscience, and; 4 Department of Ophthalmology and Visual Sciences, Albert Einstein College of Medicine, Bronx, New York, USA.

**Keywords:** Neuroscience, Reproductive Biology, Cytoskeleton, Prostate cancer, Urology

## Abstract

The microtubule (MT) cytoskeleton plays a critical role in axon growth and guidance. Here, we identify the MT-severing enzyme fidgetin-like 2 (FL2) as a negative regulator of axon regeneration and a therapeutic target for promoting nerve regeneration after injury. Genetic knockout of FL2 in cultured adult dorsal root ganglion neurons resulted in longer axons and attenuated growth cone retraction in response to inhibitory molecules. Given the axonal growth-promoting effects of FL2 depletion in vitro, we tested whether FL2 could be targeted to promote regeneration in a rodent model of cavernous nerve (CN) injury. The CNs are parasympathetic nerves that regulate blood flow to the penis, which are commonly damaged during radical prostatectomy (RP), resulting in erectile dysfunction (ED). Application of FL2-siRNA after CN injury significantly enhanced functional nerve recovery. Remarkably, following bilateral nerve transection, visible and functional nerve regeneration was observed in 7 out of 8 animals treated with FL2-siRNA, while no control-treated animals exhibited regeneration. These studies identify FL2 as a promising therapeutic target for enhancing regeneration after peripheral nerve injury and for mitigating neurogenic ED after RP — a condition for which, at present, only poor treatment options exist.

## Introduction

The microtubule (MT) cytoskeleton is a major regulator of axon growth and guidance. It serves as the major track for vesicular transport through the axon shaft and provides structural support to the axon, and in the distal region of the axon, polymerizing MTs play a critical role in promoting axon elongation and in steering the growth cone in response to environmental cues (1, 2). Given its role in regulating axonal growth, targeting the MT cytoskeleton to enhance axon regeneration after nerve injury has been proposed as a potential therapeutic strategy (3–11). This proposition is supported by studies demonstrating that modulation of MT dynamics in the axon can promote regeneration and attenuate degeneration: for example, multiple studies have found that low doses of MT-stabilizing drugs (taxol and epothilones) promote axon regeneration and improve recovery of locomotor function after spinal cord injury in rats (5–7, 11). Increasing the density of dynamic MTs in the axon by knocking down the MT-severing enzyme fidgetin was also shown to promote axon regrowth after dorsal root crush in rats (9).

MT-severing enzymes, which are members of the ATPases associated with diverse cellular activities superfamily, cause breakages in MTs by forming hexameric rings around the C-terminal tails of tubulin and using energy from ATP hydrolysis to pull on the tails, thereby causing tubulin dimers to dissociate from the MT lattice (12, 13). Through their severing activity, they regulate MT length, number, and branching and fine-tune the dynamics of the MT cytoskeleton (14, 15). MT-severing enzymes include katanin, spastin, and the fidgetin family: fidgetin, fidgetin-like 1, and fidgetin-like 2 (FL2). With the exception of FL2, all have been reported to play important roles in regulating axonal growth through their remodeling of the axonal MT array (9, 16–21).

We recently identified FL2 as a negative regulator of cell migration and a potential therapeutic target for promoting healing of cutaneous wounds: application of nanoparticle-encapsulated FL2-siRNA to murine cutaneous burn and punch biopsy wounds significantly enhanced the rate of wound closure (22, 23). In vitro, the acceleration of cell motility observed with FL2 knockdown was accompanied by an increase in the density of dynamic MTs, particularly at the leading edge of the cell, where FL2 was shown to strongly localize (22). It was therefore proposed that the enzyme normally suppresses forward movement of the cell by selectively paring down dynamic MTs near the cell cortex. Beyond these 2 studies, FL2’s functions at both the cellular and physiological level are unknown.

The goals of the present study were to characterize the role of FL2 in regulating axonal regeneration and assess its potential as a therapeutic target for promoting regeneration after nerve injury. For the latter, we utilized a rodent model of peripheral nerve damage in which the cavernous nerves (CNs) of rats were either crushed or transected, mimicking CN damage associated with radical prostatectomy (RP) (24, 25). The CNs are parasympathetic nerves that travel along the posterolateral prostate and innervate the penis, where they regulate erectile function by controlling blood flow to the corporal tissue. Because they lie on the prostate, they are highly susceptible to damage during RP, resulting in erectile dysfunction (ED) (26).

This model is highly clinically relevant, since currently only poor treatment options exist for CN injury–induced ED after RP (27). Prostate cancer is the second most prevalent cancer in men (28), and RP is the most common treatment for localized prostate cancer, particularly for younger men who are sexually active (29). Unfortunately, even with the advent of nerve-sparing procedures, incidences of ED after RP are high: 60% of patients experienced self-reported ED 18 months after RP, and only 28% of men reported erections firm enough for intercourse at a 5-year follow-up according to a prostate cancer outcomes study (27, 30). ED after RP has a major impact on the lives of many patients and their partners.

We demonstrate that FL2 is a negative regulator of axon regeneration that appears to suppress axonal growth by selectively severing dynamic MTs in the distal axon shaft and growth cone. We further show that FL2 depletion attenuates the effects of inhibitory environmental cues on growth cone advancement. Finally, we demonstrate that targeted knockdown of FL2 after CN injury is effective in promoting regeneration and restoration of nerve function in rats. These observations suggest a novel therapeutic approach to treat peripheral nerve injury, particularly injuries resulting from surgical procedures such as RP, where treatments depleting FL2 could be applied locally at the time of injury.

## Results

### FL2 suppresses axonal growth in dissociated adult dorsal root ganglion neurons.

**Figure 1:**
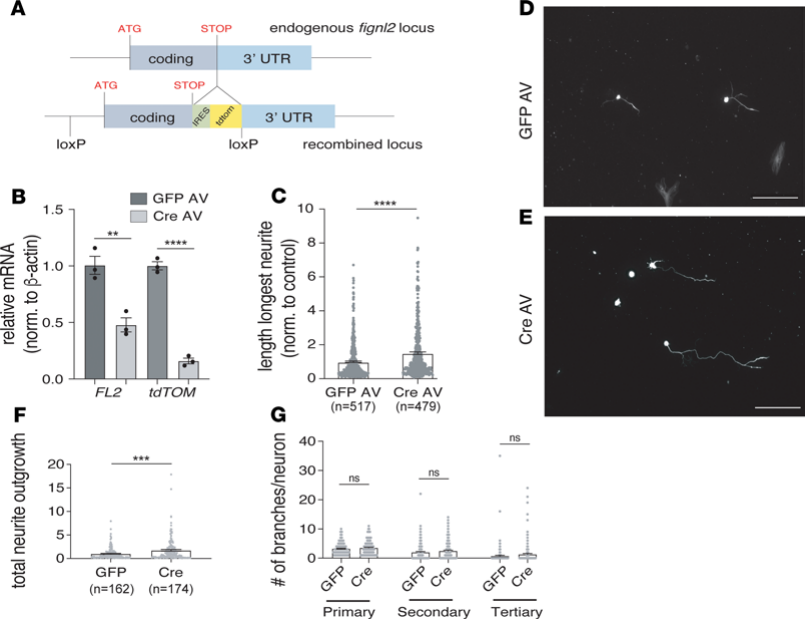
FL2 depletion accelerates the rate of axon regeneration in adult DRG neurons. (**A**) Schematic of internal ribosome entry site–tdTomato (IRES-tdTomato) knockin and *LoxP* sites at *Fignl2* endogenous locus. The *Fignl2* gene is on chromosome 15 and is intronless, composed of 1 exon extending over 4.2 kB. An IRES and *tdtomato* (*tdTOM*) reporter gene were inserted after the stop codon between the coding sequence and 3′ UTR sequence. *LoxP* sites were inserted upstream of the start codon and after the reporter gene. (**B**) *Fignl2* and *tdTOM* mRNA levels 1 week after transduction with GFP AV or Cre AV. RNA combined from 4 transduced cultures for each experiment. (**C**) Average length of longest neurites in control and FL2-knockout neurons 2 days after replating (GFP AV mean ± SEM: 1.00 ± 0.05; Cre AV: 1.51 ± 0.07. GFP AV *n* = 517, Cre AV *n* = 479). Experiment performed in quadruplicate. (**D** and **E**) Micrographs of GFP AV (**D**) and Cre AV (**E**) DRG neurons replated at low density 1 week after viral transduction, fixed 2 days later, and immunostained for tubulin (tubulin isoform β3) (scale bar: 200 μm). (**F**) Neurite outgrowth (sum length of all neurites per neuron) in GFP AV– and Cre AV–treated neurons 2 days after replating, normalized to the control mean (GFP AV mean ± SEM: 1.00 ± 0.10; Cre AV: 1.74 ± 0.18). GFP *n* = 162, Cre AV *n* = 174. (**G**) Numbers of primary, secondary, and tertiary neurites on GFP AV– and Cre AV–treated neurons (primary: GFP AV: 3.26 ± 016, Cre AV: 3.55 ± 0.18, *P* = 0.24, secondary: GFP AV: 2.01 ± 0.24, Cre AV: 2.47 ± 0.24, *P* = 0.17; tertiary: GFP AV: 0.72 ± 0.25; Cre AV: 1.3 ± 0.27, *P* = 0.12. GFP *n* = 159, Cre AV *n* = 173). Experiments performed in triplicate unless otherwise noted. Data were analyzed using unpaired 2-tailed Welch’s *t* test (morphometric data) or 2-tailed Student’s *t* test (qPCR data). Bars represent mean ± SEM. **P < 0.01; ***P < 0.001; ****P < 0.0001.

An FL2 conditional knockout (KO) mouse with a tdTomato reporter gene insertion was generated by genOway (Figure 1A and Supplemental Figures 1–5; supplemental material available online with this article; https://doi.org/10.1172/jci.insight.138484DS1; see Methods for more information). To test whether FL2 regulates axon growth, adult dorsal root ganglion (DRG) neurons harvested from FL2-flox homozygous mice were transduced ex vivo with adenovirus (AV) containing either a Cre recombinase plasmid (Cre AV) to excise the FL2 gene or GFP control plasmid (GFP AV). The neurons were cultured for 1 week to enable sufficient time for FL2 depletion following Cre-mediated excision of the gene. Transduction efficiency was approximately 80% based on the percentage of GFP-positive control neurons 5 days after transduction. Because no antibody against rodent FL2 exists, successful gene excision was confirmed by quantification of FL2 and tdTomato mRNA levels 1 week after transduction by reverse transcription quantitative polymerase chain reaction (RT-qPCR) (Figure 1B). One week after transduction, neurons were replated at low density to allow neurite growth to begin anew and fixed 48 hours later for morphometric analysis. FL2-depleted neurons had significantly longer axons (indicated by the length of the longest neurite on each neuron) compared with controls, with a mean axon length approximately 50% longer than GFP AV–treated neurons (*P* < 0.0001, Welch’s *t* test) (Figure 1, C–E). These results were consistent with preliminary studies we conducted in rat neurons transduced with plasmids encoding scrambled or FL2-shRNA and replated for regrowth (Supplemental Figure 6). We observed that cell densities prior to and after replating were similar between GFP AV– and Cre AV–treated neurons, indicating FL2 depletion did not significantly impact cell survival; however, no quantitative assays assessing cell viability were performed.

MT remodeling is critical for neurite initiation and neurite branching (1), and the MT-severing enzyme spastin was found to regulate axonal branching (17). We therefore also quantified total neurite outgrowth and branching by tracing all primary, secondary, and tertiary neurites in a smaller subset of neurons from 3 of the experiments (images were selected sequentially from each treatment group for tracing until 50–60 neurons per experiment were quantified). Consistent with the increase in the length of the longest axon, the sum length of neurite outgrowth was significantly increased in Cre AV–treated neurons compared with controls (Figure 1F). The average numbers of primary, secondary, and tertiary neurites for these neurons, however, were not significantly altered (Figure 1G). There was a trend toward an increased number of branch points with Cre AV treatment (represented by the average number of secondary and tertiary neurites, Figure 1G); however, this trend is likely a secondary effect due to the significantly increased length of primary neurites, since neurons with longer primary neurites had a greater likelihood of branching in both treatment groups (Supplemental Figure 7).

### FL2 depletion results in a more dynamic MT array in the distal axon.

Axonal MTs are composed of domains that differ markedly in their stability properties: there are a fraction of labile, highly dynamic MTs; a fraction of stable, long-lived, and relatively undynamic MTs; as well as a hyperstable fraction of polyaminated MTs (referred to as the cold-stable fraction) (31, 32). Longer lived (stable) MTs accumulate posttranslational modifications, such as acetylation, polyglutamylation, and de-tyrosination, while labile MTs are relatively unmodified (33). It has been hypothesized that an increase in the density of labile, unmodified MTs in the distal axon is conducive to axon regeneration (31, 34, 35), and MT-severing enzymes are known to selectively target different MT subpopulations within the axon. For example, katanin and spastin preferentially target stable MTs (36, 37), while fidgetin severs dynamic MTs (20). We therefore characterized changes in the relative ratios of dynamic and stable MTs in DRG neurites with FL2 depletion.

**Figure 2:**
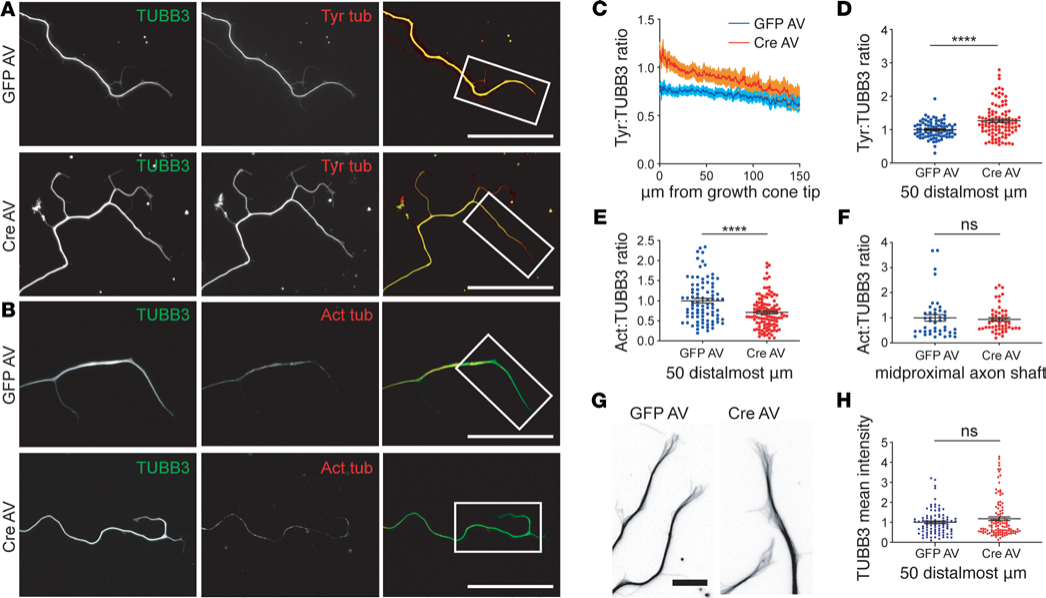
FL2 depletion results in a more dynamic MT array near the growth cone in regenerating neurites. (**A** and **B**) Immuno-micrographs of DRG neurites of GFP AV– and Cre AV–treated neurons 2 days after replating, dual-stained for tubulin isoform β3 (TUBB3) (green) and tyrosinated tubulin (Tyr tub) (red) (**A**) or for TUBB3 (green) and acetylated tubulin (Act tub) (red). Brightness/contrast adjusted linearly to more clearly see acetylated tubulin stain (**B**). Scale bars: 50 μm. (**C**) Tyr/TUBB3 ratios in GFP AV– and Cre AV–treated neurons from 1 representative experiment, starting at the tip of the growth cone and moving toward the soma (dark line is the mean intensity ratio; shaded area represents the SEM). (**D**) Tyr/TUBB3 fluorescence intensity ratios in the 50 distalmost micrometers of neurites, normalized to the GFP AV mean (GFP AV: 1.00 ± 0.03, Cre AV: 1.26 ± 0.05, *n* = 86 GFP, *n* = 101 Cre). (**E**) Distribution of Act/TUBB3 ratios in the 50 distalmost micrometers of neurites, normalized to the GFP AV mean (GFP AV: 1 ± 0.06, Cre AV: 0.74 ± 0.04. *n* = 87 GFP; *n* = 116 Cre). (**F**) Act/TUBB3 ratios in the proximal to mid region of the axonal shaft (GFP AV: 1.00 ± 0.13; Cre AV: 0.94 ± 0.07, *P* = 0.68, *n* = 43 GFP, *n* = 51 Cre). Experiment performed in duplicate. (**G**) Inverted images of MTs in the growth cones (GCs) of GFP AV– and CRE AV–treated neurons, immunostained for TUBB3. Brightness/contrast adjusted to better visualize individual MTs. Scale bar: 10 μm. (**H**) Mean TUBB3 fluorescence intensities in the distalmost 50 μm of neurites (GFP: 1.00 ± 0.06, Cre: 1.18 ± 0.09, Welch’s *t* test, *P* = 0.12, *n* = 91 GFP, *n* = 101 Cre). Experiments performed in triplicate unless otherwise indicated. Data were analyzed using unpaired 2-tailed Welch’s *t* test. Bars represent mean ± SEM. ****P < 0.0001.

To characterize changes in the axonal MT array with FL2 depletion, we measured changes in the ratios of dynamic and stable MTs to total MTs along neurites by quantitative immunofluorescence. Neurons were dual-stained with either tyrosinated tubulin and the β-tubulin isoform TUBB3 or acetylated and TUBB3 (tyrosinated and acetylated tubulin are commonly used as markers of labile and stable MTs, respectively, while TUBB3 is a neuron-specific isoform of tubulin that is present in both stable and labile MTs). We found that FL2-depleted neurons had significantly higher ratios of tyrosinated to TUBB3 and, conversely, lower ratios of acetylated to βIII tubulin in the distalmost region of neurites: the tyrosinated/TUBB3 ratio increased by approximately 26% while the acetylated/TUBB3 ratio correspondingly decreased by approximately 26% in the distalmost 50 μm of neurites (*P* < 0.0001, Welch’s *t* test) (Figure 2, A–E). The average TUBB3 fluorescence intensity in the distalmost 50 μm of neurites was not significantly different between GFP and Cre AV–treated neurons (Figure 2H), indicating the total density of MTs did not differ significantly although the ratios of dynamic and stable MTs in this region had shifted. The ratio of acetylated/TUBB3 in the midproximal neurite shaft was unaffected by FL2 knockout (Figure 2F), suggesting that the enzyme’s activity and/or localization may be most concentrated in (or restricted to) the distal axon and growth cone.

We therefore more closely examined the organization of the MT array in the growth cone (GC) as well as GC morphology in neurons dual-stained for TUBB3 and actin (Figure 2G and Supplemental Figure 8). In fixed cells, we did not detect any differences in the morphology of the GC MT array (such as increased MT splay, increased MT invasion of filopodia, buckling, or looping) with FL2 knockout, and GC size, filopodia number, and filopodia length also appeared unaffected by FL2 depletion (Supplemental Figure 8).

### FL2 mediates GC response to inhibitory environmental cues.

**Figure 3:**
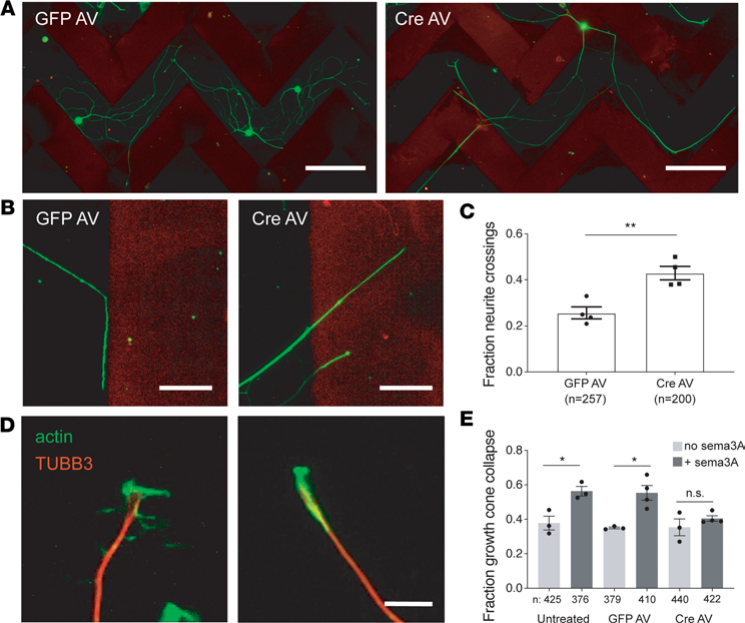
FL2 depletion attenuates the effects of inhibitory substrates on adult DRG GC advancement during regeneration. (**A**) Micrographs of GFP AV– and Cre AV–treated neurons immunostained for TUBB3 (green) 72 hours after plating on aggrecan stripes (red). Scale bar: 200 μm. (**B**) High-magnification image of a GFP AV–treated neurite turning in response to an aggrecan border and Cre AV–treated neurites crossing through the stripe border. Scale bar: 50 μm. (**C**) Fraction of neurites of GFP AV– and Cre AV–treated neurons that crossed aggrecan stripe borders (GFP AV mean = 0.26 ± 0.026; Cre AV mean = 0.41 ± 0.06. *P* = 0.0169, unpaired 2-tailed Welch’s *t* test, GFP *n* = 65 neurons, 257 neurite/border encounters; Cre AV *n* = 79 neurons, 200 neurite/border encounters. Experiment performed 4 times). (**D**) Immuno-micrographs showing an active, viable GC (left) and a collapsed GC (right), stained for MTs (TUBB3) in red and actin in green. Scale bar: 10 μm. (**E**) Fraction of collapsed GCs in untreated, GFP AV–treated, and Cre AV–treated neurons, in the presence and absence of Sema3A. GFP and Cre AV experiments performed in quadruplicate, untreated +/– Sema3A performed in triplicate (untreated, no Sema3A: 0.38 ± 0.04, *n* = 425 GCs; untreated, +Sema3A: 0.56 ± 0.03, *n* = 376; GFP AV, no Sema3A: 0.35 ± 0.01, *n* = 379; GFP AV, +Sema3A: 0.55 ± 0.04, *n* = 410; Cre AV, no Sema3A: 0.35 ± 0.05, *n* = 440; Cre AV, +Sema3A: 0.40 ± 0.02, *n* = 422. Mean ± SEM; *P < 0.05, **P < 0.01; unpaired 2-tailed Welch’s *t* test). Imaging and analyses performed in a blinded manner.

Given the evidence that FL2 was suppressing axonal growth by severing dynamic MTs in the distalmost axon and growth cone, we hypothesized that FL2 might also mediate growth cone response to environmental cues. To test this, we first challenged regenerating axons with a mixture of chondroitin sulfate proteoglycans (CSPGs). CSPGs are abundant in the glial scar tissue at central nervous system (CNS) injury sites and impede axon regeneration through the scar by causing GCs to retract (38). We used a modified version of a conventional stripe assay (39) in which neurons were replated onto stripes of CSPG mixed with Alexa Fluor 568–anti-mouse IgG antibody (in order to visualize the stripe boundaries). The majority of adult DRG neurons turn upon encounter with these aggrecan stripes, whereas stripes of Alexa Fluor 568–anti-mouse IgG alone elicit no turning response from GCs. After 3–4 days, the neurons were fixed and imaged, and the percentage of neurites crossing the stripe borders out of total neurite/border encounters was quantified. FL2-depleted neurons had a significantly higher percentage of neurites crossing through stripe borders compared with GFP AV–treated control neurons (43% ± 3% versus 26% ± 4%) (Figure 3, A–C).

In a complementary experiment, neurons were challenged with recombinant semaphorin 3A (Sema3A). Sema3A is an important chemorepellent during the development of the nervous system (40, 41) and has been implicated in axon guidance during adult PNS regeneration (42–44). In vitro, Sema3A causes GC collapse in both embryonic and adult DRG neurons (45). In adult DRG neurons, the Sema3A-induced GC collapse phenotype is less pronounced; this is because adult DRG neurons have smaller GCs that are more likely to assume a bullet shape — meaning a higher percentage of GCs exhibit a “collapsed” phenotype to begin with (Figure 3D) — and, additionally, response to Sema3A is limited in adult DRGs to nociceptive neurons (which make up the majority of small-bodied DRG neurons), whereas in embryonic DRGs the response to Sema3A is ubiquitous (46).

We first tested whether Sema3A induced GC collapse in our 9-day-old cultures of adult DRG neurons, 2 days after replating. For GCs to be counted as collapsed, GC MTs had to be bundled and no filopodia could be present (the width of these collapsed GCs was less than 2 μm). We found that treatment with a recombinant Sema3A-Fc chimera caused an approximately 20% increase in the percentage of GCs exhibiting a collapsed phenotype compared with neurons treated with Fc alone (Figure 3E), consistent with a previous study, which found that GCs of adult rat DRG neurons with a collapsed phenotype rose 18% with Sema3A treatment in vitro (46). Similarly, GFP AV–treated neurons showed an approximately 20% increase in GC collapse with Sema3A treatment (Figure 3E). In contrast, FL2-depleted neurons showed no significant increase in GC collapse in the presence of Sema3A (Figure 3E). Together, these results indicate that FL2 plays an important role in mediating GC response to inhibitory substrates in adult sensory neurons.

### Targeted downregulation of FL2 following CN crush promotes functional nerve regeneration.

Based on the growth-promoting effects of FL2 depletion on regenerating axons in vitro, we reasoned that using siRNA to knock down expression of FL2 at the site of nerve injury would promote nerve regeneration and recovery of nerve function. To test this hypothesis, an animal model of nerve injury was used in which the CNs are crushed, mimicking CN damage and consequent neurogenic ED caused during RP (24, 25).

**Figure 4:**
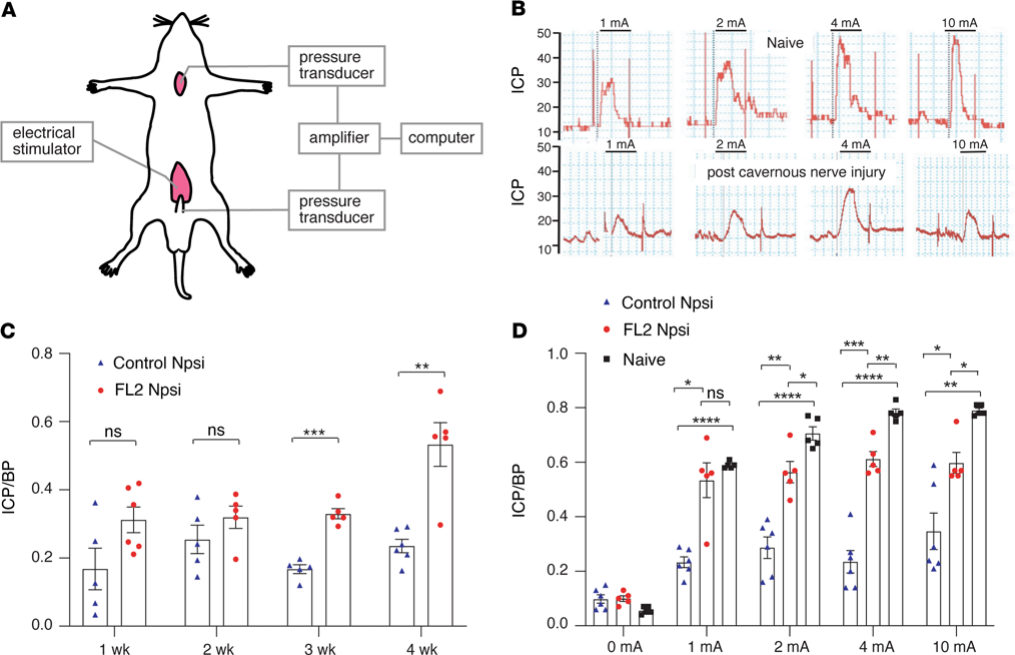
Treatment with nanoparticle-encapsulated FL2-siRNA improves recovery of erectile response following CN injury. (**A**) Schematic of cavernosometry setup. Cannulas are inserted into the penile cruz and the carotid artery to measure the ICP and BP, respectively. A bipolar stainless steel electrode, inserted above the site of nerve injury, is used to directly stimulate the CN. (**B**) Traces of the ICP in response to increasing levels of CN stimulation in an uninjured animal and an animal following cavernous nerve injury. (**C**) Time course showing the ratio of ICP to mean BP (ICP/BP) of control-npsi– and FL2-npsi– treated nerves with 1 mA electrostimulation of the nerve (5–6 animals per time point and treatment group — note the experiment is terminal and a different cohort was used for each time point). (**D**) ICP/BP for naive and npsi-treated rats 4 weeks after crush and treatment (Control Npsi *n* = 6; FL2 Npsi *n* = 5; naive *n* = 5). Data are presented as mean ± SEM and were analyzed using unpaired 2-tailed Student’s *t* test (**C**) or 1-way ANOVA with Tukey’s test (**D**). *P < 0.05; **P < 0.01; ***P < 0.001; ****P < 0.0001.

In these experiments, we pooled 3 siRNA sequences targeting rat FL2 mRNA and encapsulated the siRNA into a nanoparticle formulation that has been used previously in other preclinical models to deliver siRNA to wounds (22, 47). Efficacy of the formulation was confirmed in vitro in B35 cells (a rat neuroblastoma cell line) (Supplemental Figure 9). Ten microliters of a 10 μM suspension of nanoparticle-encapsulated FL2-siRNA (FL2-npsi) or scrambled-siRNA (control-npsi) was pipetted onto the CN and the major pelvic ganglia (MPG) immediately after nerve crush (the CNs on both sides of the posterolateral prostate were crushed for 2 minutes using smooth forceps, 2–3 mm from where the nerves emanate from the MPG). Functional nerve regeneration was assessed by performing cavernosometry, wherein CNs are electrically stimulated above the injury site and increases in intracorporal pressure (ICP) in response to nerve stimulation are measured (Figure 4, A and B). The increase in ICP in response to nerve stimulation is mediated by the release of nitric oxide (NO) from CN nitrergic axons into the corpus cavernosum (48). This release of NO into the corpus cavernosum is the primary initiator of the erectile response (49).

A time course for functional CN regeneration was determined by cavernosometry performed weekly over a month. At the 3- and 4-week postinjury time points, cavernosometry demonstrated the FL2-npsi–treated animals had significantly improved erectile function compared with animals treated with control-npsi (Figure 4, C and D). At 4 weeks after, the ICP/BP response of FL2-npsi–treated nerves was comparable to that of naive animals at low levels of stimulation (Figure 4D).

### FL2 knockdown after CN crush increases the density of nitrergic neurons in the penis.

**Figure 5:**
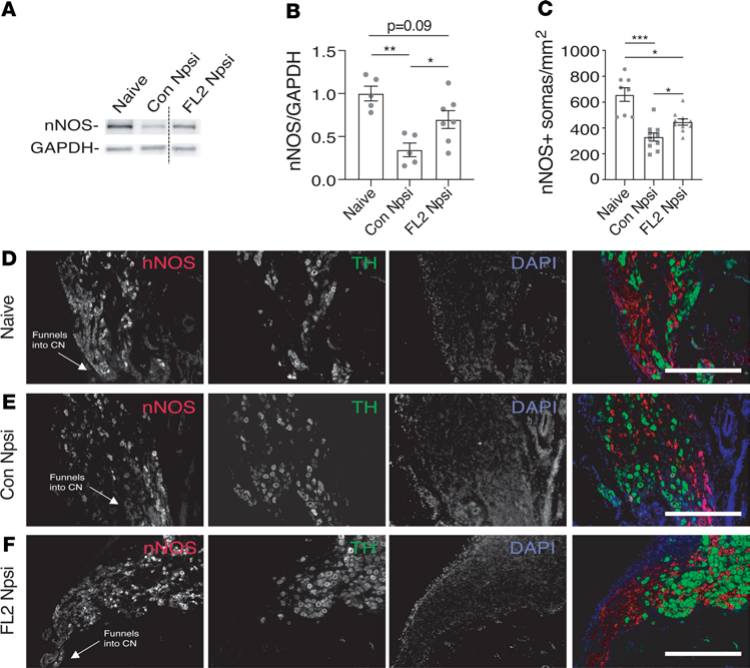
FL2-npsi–treated nerves had increased nNOS in the penis shaft and an increased density of nitrergic neurons in the caudal MPG 4 weeks after CN crush. (**A**) Representative lanes from Western blot of penile shaft samples from naive animals and from rats 4 weeks after 4-minute CN crush and treatment with control- or FL2-npsi, probed for nNOS and GAPDH (line divides noncontiguous lanes on the same blot). (**B**) Relative levels of nNOS in the penile shaft analyzed by Western blot and normalized to GAPDH, 4 weeks after CN crush and treatment with FL2- or control-npsi (naive: 1.0 ± 0.09, *n* = 5; Con Npsi: 0.35 ± 0.08, *n* = 5; FL2 Npsi: 0.70 ± 0.10, *n* = 7). One-way ANOVA with Tukey’s correction. (**C**) Average density of nNOS^+^ somas proximal to the CN in naive and control- and FL2-npsi–treated animals 4 weeks after CN crush (naive: 655.2 ± 15.8; Con Npsi: 335.4 ± 34.7; FL2 Npsi: 451.7 ± 30.9). Mean ± SEM; *P < 0.05, **P < 0.01, ***P < 0.001; Brown-Forsythe and Welch’s ANOVA with Dunnett’s T3 correction. Eight to 10 sections from 3–4 MPG analyzed per treatment group. (**D**–**F**) Images of longitudinal sections of MPG proximal to the CN from a naive animal (**D**) and from animals 4 weeks after CN crush and treatment with control-npsi (**E**) or FL2-npsi (**F**) stained for nNOS to label nitrergic neurons and tyrosine hydroxylase as a counterstain to label sympathetic noradrenergic neurons and DAPI (scale bar: 0.5 mm).

To confirm the regenerative effect of downregulating FL2, we assessed levels of neuronal nitric oxide synthase (nNOS) in the penile shaft by Western blot 1 month following CN crush and treatment (nNOS is present in the axons of nitrergic neurons) (50–52). Consistent with the electrophysiology data, we found significantly higher levels of nNOS in the penes of FL2-npsi–treated animals compared with controls, indicating enhanced reinnervation of the penile shaft by regenerated nitrergic neurons with FL2-npsi treatment (Figure 5, A and B).

### FL2 knockdown after CN crush increases the density of nitrergic neurons in the caudal MPG.

Previous work has shown that proapoptotic genes are activated in the MPG 48 hours to 14 days after CN crush (53) and that apoptosis of neurons in the MPG peaks 7–14 days after injury (54). To test whether FL2-npsi also improved neuronal survival, we immunostained the MPG and quantified the density of somas positive for nNOS in the caudal region of the MPG. Consistent with the increase in nNOS in the corporal tissue, we found the density of nitrergic neurons in the caudal MPG was also increased (Figure 5, C–F).

### Targeted knockdown of FL2 after CN transection promotes functional nerve regrowth.

**Figure 6:**
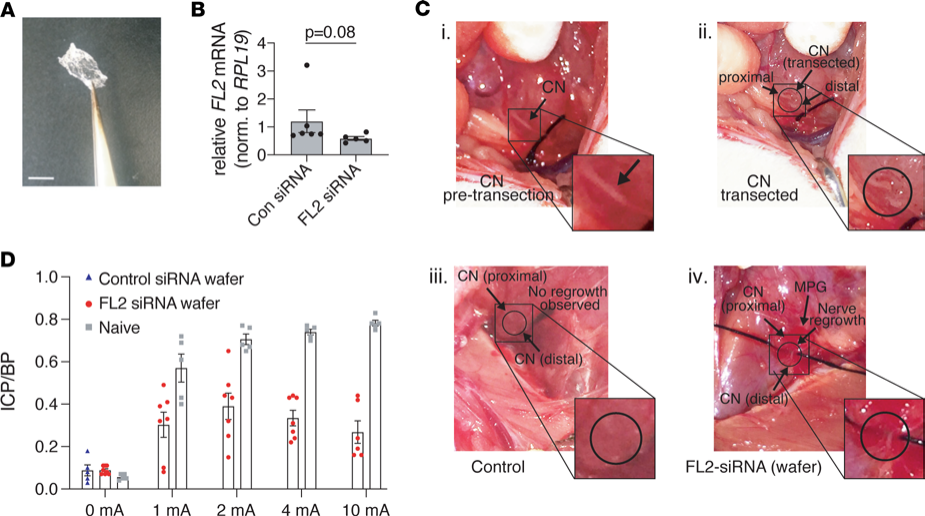
Application of FL2-siRNA after bilateral CN transection leads to visible regeneration and partial recovery of erectile function. (**A**) Image of siRNA wafer prior to application. Scale bar: 0.5 cm. (**B**) Relative levels of *FL2* mRNA in the MPG 2 weeks after CN transection and wafer treatment, normalized to ribosomal protein L19 (*RPL19*) (unpaired 2-tailed Student’s *t* test, *P* = 0.08, *n* = 5). (**C**) Images of the CN prior to transection (i); immediately after transection (ii); and 2 weeks after transection and con-siRNA or FL2-siRNA wafer treatment (iii and iv, respectively). (**D**) Mean maximal ICP/BP measurements following different levels of stimulation of transected/siRNA wafer–treated animals and naive age-matched controls (mean ± SEM). Note control-siRNA wafer transected nerves could not be stimulated due to the degree of retraction of the severed nerve segments; therefore only baseline ICP/BP is shown.

We then tested whether FL2 downregulation could be effective in promoting nerve regeneration after a more severe injury. The CNs were transected bilaterally, resulting in a gap of 3–4 mm between the proximal and distal nerve segments. For these experiments, we had the FL2- or scrambled-siRNA embedded into hardened chondroitin sulfate microgels (Figure 6A), which adhere to tissue upon application and slowly dissolve onto the injury site, better confining the treatment to the injury site than the liquid nanoparticle suspension. A 0.4 × 0.8 cm piece of wafer was applied immediately after transection, covering the injury site, the entire MPG, and some of the surrounding prostate tissue. We were unable to determine knockdown efficiency in vivo following treatment with either FL2-npsi or wafers, which we suspect may be due to the varied abundance of FL2 mRNA in different cell types in the tissue sample (both the MPG and proximal CN were harvested), as well as the overall low abundance of FL2 mRNA in the tissue sample. We did, however, observe a trend toward reduced FL2 expression in the MPG of animals treated with FL2-siRNA wafer 14 days after treatment (approximately 43% lower levels) (Figure 6B).

Two weeks following treatment of CN-transected animals with FL2-siRNA wafer, 7 out of 8 animals exhibited visible CN regeneration, whereas in animals treated with control-siRNA wafers, the 2 severed nerve segments had further retracted from each other, and there was no visible nerve regrowth (Fisher’s exact test, *P* = 0.0004) (Figure 6C). In animals with visible regeneration of the CN, cavernosometry was performed. Cavernosometry could not be performed on any control animals since there was no nerve to stimulate. At all levels of electrostimulation, animals treated with FL2-siRNA wafer had a significant erectile response, as evidenced by an increase in the ICP/BP over basal (unstimulated) ICP/BP (Figure 6D), indicating some regenerated axons had reached the target tissue. The maximal ICP/BP reached was at the 2 mA level of stimulation and was 55% of the erectile response achieved when naive age-matched control animals were stimulated at the same level. At higher levels of stimulation than 2 mA, both the maximal erectile response and percentage response relative to naive animals decreased.

**Figure 7:**
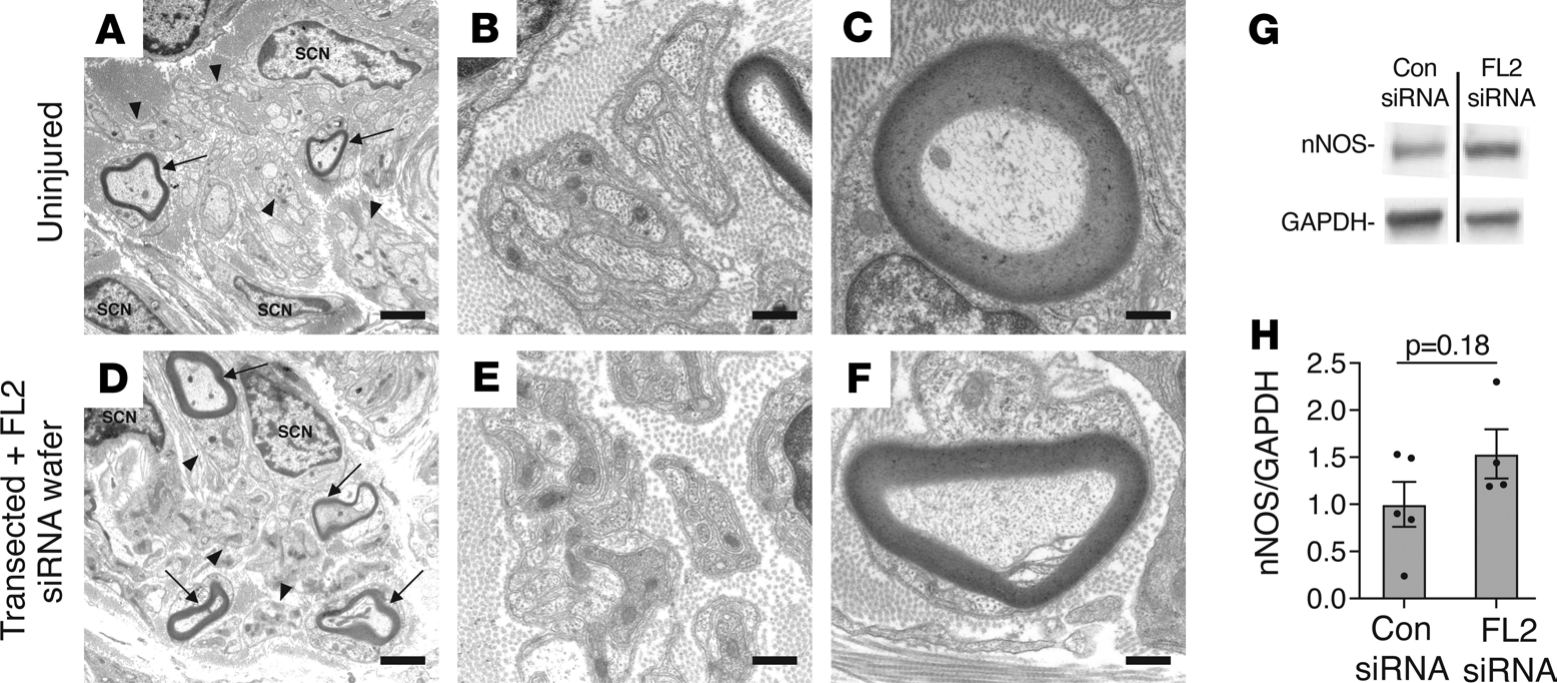
Regenerated myelinated and unmyelinated axons are present in the CN distal to the injury site following transection and FL2-siRNA wafer treatment. (**A** and **D**) Transmission electron microscopy (TEM) images, original magnification, ×5000, of a distal segment of a CN from an uninjured animal (**A**) and 1 from a transected and FL2-siRNA wafer–treated animal (**D**), 4 weeks after transection and treatment (harvest of control-siRNA wafer–treated distal nerve segments was not possible due to retraction of the distal nerve segment). Arrows point out myelinated large-diameter axons. Arrowheads point to some of the Remak bundles of unmyelinated small-diameter axons. Scale bar: 2 μm. SCN, Schwann cell nucleus. (**B** and **E**) TEM images, original magnification, ×20,000, of an uninjured (**B**) and transected and FL2-siRNA wafer–treated nerve (**E**) showing Remak bundles at higher magnification. Scale bar: 0.5 μm. (**C** and **F**) ×20,000 TEM images of uninjured CN (**C**) and a transected and FL2 siRNA wafer–treated nerve distal to injury site (**F**), showing myelinated axons. Scale bar: 0.5 μm. (**G**) Representative lanes from Western blot of corporal tissue lysates from control and FL2-siRNA wafer–treated animals, 1 month following bilateral transection of the CN, probed for nNOS and GAPDH (lanes rune on same gel but noncontiguous). (**H**) Relative levels of nNOS in the penile shaft analyzed by Western blot and normalized to GAPDH, 4 weeks after bilateral CN transection and treatment with FL2- or control-siRNA wafers. Only FL2-siRNA wafer–treated animals that exhibited regeneration in both CNs included in analysis. Control mean ± SEM = 1.00 ± 0.24, FL2 si = 1.5 ± 0.26, *P* = 0.18, unpaired 2-tailed Welch’s *t* test, control *n* = 5, FL2 si *n* = 4.

To confirm the presence of regenerated axons distal to the transection site following treatment, a segment of (unstimulated) regenerated nerve distal to the transection site was harvested from an FL2-siRNA wafer–treated animal 4 weeks following transection and treatment, along with a distal nerve segment from an uninjured control animal. The nerves were processed for ultrastructural analysis by transmission electron microscopy (TEM) (Figure 7, A–F). Large-diameter myelinated axons as well as Remak bundles of small-diameter unmyelinated axons were identified in cross sections of regenerated nerve tissue; however, as would be expected, both myelinated axons and Remak bundles were sparser, and Remak bundles contained fewer axons than those observed in the uninjured nerve at this time point (Figure 7, B and E). We observed no qualitative difference in the myelin sheaths of regenerated and uninjured nerve tissue, but further study is required to determine whether FL2 downregulation impacts remyelination. It was not possible to harvest a control-siRNA–treated nerve segment distal to the injury site because of the degree to which the nerve segment had retracted.

To examine the extent to which target reinnervation occurred with FL2-siRNA wafer treatment, an additional cohort of animals (*n* = 6 per treatment group) underwent transection and wafer treatment for analysis of the corporal tissue 1 month after injury and treatment. In this group 4 of the FL2-siRNA wafer–treated animals showed visible regeneration of the nerve sheath bilaterally, 1 animal showed regrowth only on 1 side, and 1 animal showed no regrowth on either side. No control animals exhibited successful repair of the nerve bridge area on either side of the prostate. Recovery of nNOS in the corporal tissue at 1 month was less clear than in the crush study, but Western blot of penile shaft lysates from control animals and from the FL2-siRNA wafer–treated animals that exhibited bilateral CN regeneration showed a trend toward increased levels of nNOS (Figure 7, G and H).

## Discussion

To our knowledge, this is the first report identifying FL2 as a negative regulator of axonal growth. Our data suggest that the enzyme suppresses axonal growth and mediates GC response to some environmental cues through its presumptive severing activity in the GC and distal axon. Furthermore, we have shown in vivo that targeted knockdown of FL2, through the application of FL2-siRNA, promotes peripheral nerve regeneration and recovery of nerve function.

While we are unable to confirm the subcellular localization of FL2 in rodent neurons by immunostaining, the phenotype observed here — taken together with FL2’s previously reported cortical localization and role as a negative regulator of cell motility in human cells (22) — suggests the enzyme may function similarly to cortical collapse factors (cell surface–associated MT-regulatory proteins that inhibit MT polymerization at the cortex) (55). For example, in some species, the cortical collapse factor Efa6 negatively regulates axonal growth through its inhibition of MT polymerization in the GC (55), and mammalian KIF21α also negatively regulates axonal growth through inhibition of MT polymerization near the cortex (56). Regulation of cortical MTs by FL2 during cell motility and GC advancement may be especially critical during development, when cells and axons must navigate complex environments and identify their appropriate destinations. While FL2’s function has yet to be studied during development, a recent study of the expression patterns of the fidgetin proteins during zebrafish development showed that FL2 is highly expressed in the developing CNS as well as many other tissues, including the somites, eyes, pectoral fins, and pronephros (57).

The observed increase in the average ratio of dynamic to total MTs in the distalmost segment of DRG neurites with FL2 depletion is consistent with other studies that indicate that a more dynamic MT array in the distal axon is conducive to axon growth. For example, MT deacetylation has been shown to be critical to the regeneration of adult peripheral neurons after injury (such a shift does not occur in injured CNS neurons, which lack the regenerative capacity of their PNS counterparts) (34), and dynamic MTs are critical for reformation of a viable GC (2, 58–60). Notably, depletion of fidgetin — FL2’s closest structural relative — increases labile MT density in the axon and has been shown to enhance axonal growth (8, 9, 20). Further work is required to fully understand how FL2 remodels the axonal MT array and whether fidgetin and FL2 have overlapping roles within the axon or regulate axonal growth through distinct mechanisms. Interestingly, while both fidgetin and FL2 suppress axonal growth, they appear to exert opposing effects in migrating cells: knockdown of fidgetin in astrocytes was found to decrease cell motility (61), whereas knockdown of FL2 increases directional cell motility in several cell types (22), demonstrating that the enzymes have distinct roles in some cellular processes.

The fact that FL2 depletion attenuates the effects of inhibitory cues on GC advancement indicates the enzyme could be a promising therapeutic target for promoting axon growth in the CNS as well, where a major impediment to axon regeneration is the abundance of inhibitory molecules at the injury site (60, 62). Axons in which FL2 has been depleted may be better able to traverse the glial scar after CNS injury. Inhibitory substrates have not been shown to be a major limitation in PNS regeneration; however, some studies indicate that semaphorins influence PNS axon regeneration and target reinnervation (42, 43, 45, 63, 64). Interestingly, Nangle and Keast demonstrated that adult parasympathetic and sympathetic neurons from the rat pelvic ganglia exhibit GC collapse in the presence of Sema3A (42), raising the possibility that Sema3A could impact the regeneration of pelvic ganglia neurons, but no studies testing this hypothesis in vivo have been conducted.

To our knowledge, regeneration across a gap of several millimeters following bilateral CN transection has not been observed without the use of a nerve graft. In studies where autologous and acellular nerve grafts were used to bridge 5 mm CN excision gaps in rats, the maximal ICP/BP achieved was 20%–25% of that of naive animals at 1 month and approximately 50% at 3 months after surgery (65). In our study, the average maximal ICP/BP reached in FL2-siRNA–treated animals was 55% that of naive age-matched control animals at 2 weeks, indicating a level of regeneration comparable and possibly superior to that seen with nerve grafts (albeit differences in the injury models and the time points at which nerve function was evaluated limit direct comparison).

From a mechanistic standpoint, while our cell biology studies identify FL2 as a negative regulator of axonal growth, the regenerative effect of FL2 depletion on the injured CN is likely multifactorial (and not the consequence of FL2 downregulation in the axons alone). In particular, the reformation of the neurolemma across the 3–4 mm gap between the transected nerve segments indicates a mechanism at work beyond accelerated axonal growth. Peripheral nerve repair is largely dependent on Schwann cells, fibroblasts, and immune cells, among others (66–68). Following transection, axons distal to the injury site degenerate and the debris is cleared from the tissue by Schwann cells and immune cells in a process called Wallerian degeneration, a process that is critical for proper axon regeneration to ensue (66). Meanwhile, Schwann cells must directionally migrate out of the nerve stumps and into the gap between the transected nerve ends, where they form corridors that guide regenerating axons into the distal nerve stump (referred to as Büngner bands) (69). The migration of Schwann cells and the formation of these corridors across the nerve bridge is orchestrated by cell signaling between the Schwann cells and fibroblasts that accumulate in the gap following injury (67). As FL2 knockdown was found to increase directional cell migration (22) and enhance wound healing in other tissues (22, 23, 47), it is possible that treatment improves formation of new nerve tissue across transection gaps by enhancing directional migration of Schwann cells or other cells at the injury site and that these effects on nonneuronal cells in turn accelerate functional axon regeneration.

Mechanistic questions notwithstanding, these studies provide clear evidence that localized downregulation of FL2 after CN injury positively impacts nerve repair and improves functional recovery. Future work should determine the effects of FL2 knockdown on other cell types involved in peripheral nerve repair, and extensively examine changes in the corpora and MPG, in order to elucidate the mechanism behind enhanced repair and functional recovery.

## Methods

### Generation of the FL2 conditional KO mouse.

The FL2 conditional KO mouse was generated by genOway. *Fignl2* is intronless, composed of 1 exon that extends over 4.2 kb on chromosome 15. Both ATG initiation and stop codons are in exon 1. Polyadenylation signal consensus sequences can be found within the predicted 3′ UTR region (2273 bp). No cDNA or expressed sequence tag related to *Fignl2* gene is described in databases. However, the National Center for Biotechnology Information database proposes 1 sequence predicted by automated computational analysis for the *Fignl2* gene, in which a noncoding 5′ UTR exon is present. The putative intron 1 would be 24 kb large. Based on a bioinformatics analysis, a targeting strategy was designed to minimize the risk of interfering with regulatory elements while inserting exogenous elements and to avoid deregulating the targeted gene. An IRES-tdTomato sequence was inserted after the stop codon in exon 1, and 2 *loxP* sites flank a region starting upstream of the exon 1 coding sequence and finishing after the reporter (Supplemental Figure 1A). After knockin, a heterozygous conditional KO line was generated. Breedings were established with C57BL/6 Flp deleter mice (genOway) to excise the neomycin selection cassette and to generate heterozygous mice carrying the neo-excised conditional KO allele. Supplemental Figure 1B illustrates the Flp excision event. Heterozygous reporter floxed animals were produced and the progeny genotyped by PCR (see Supplemental Figure 2). The recombinase-mediated excision event was then further validated by Southern blot on a subset of the PCR-positive animals (Supplemental Figure 3). Through multiple rounds of breeding, a homozygous FL2 tdT flox/flox colony was established and confirmed by both traditional PCR-based genotyping and automated qPCR-based genotyping (Supplemental Figures 4 and 5).

### Preparation of poly-d-lysine/laminin–coated coverslips for neuronal culture.

Round 12 mm #1 coverslips were cleaned with 10% HCl overnight, then washed in an ultrasonicator in distilled and deionized water 3 times (20 minutes per wash). The coverslips were then stored in 70% ethanol and air-dried in the culture plate before the coating procedure. A total of 200 μL of 100 μM poly-d-lysine (PDL) and 10 μM laminin (LAM) was applied to the dried coverslips and the culture plate incubated at 37°C for 1–2 hours. Coverslips were then washed 3 times in sterile PBS.

### Neuronal cell culture.

Dorsal root ganglia (DRGs) at the thoracic and lumbar levels were harvested from adult FL2-IRES-tdTomato flox mice between 6 and 12 weeks of age and stored in media (MEM, 5% FBS, 1% glutamax, 500 units penicillin, and 500 μg streptomycin) on ice. DRGs were cleaned (extra tissue removed), washed in MEM, and then digested in 1 mg/mL of Collagenase A (Roche, 10103578001) in MEM for 90 minutes at 37°C, followed by TrypLE (Invitrogen, Thermo Fisher Scientific) for 15 to 20 minutes at 37°C as previously described (70). The DRGs were then washed with warm media and triturated in 1 mL of media with a P1000 pipette to prepare a single-cell suspension in media. The cell suspension was transferred to the top of 2 mL of warmed 15% BSA in media and spun at 160*g* for 10 minutes to clear myelin debris. The pellet was resuspended in fresh media with supplements (20 μM fluoro-deoxyuridine, 20 μM uridine, and 100 ng/mL nerve growth factor). Cells were seeded onto PDL/LAM-coated 12 mm coverslips in a 24-well plate at a concentration of roughly 10,000–15,000 neurons per coverslip. Neurons were treated with AV containing either a GFP-encoding plasmid or a Cre recombinase–encoding plasmid (Vector Biolabs, 1060 and 1045) at a viral titer of approximately 133–200 viral particles/cell (0.2 μL of a 1 × 10^10^ PFU/mL stock per well, in 300 μL of media). One week after transduction with the virus, neurons were replated onto either PDL/LAM-coated coverslips or PDL/LAM-coated aggrecan stripes for neurite growth to begin anew. For the replating, neurons were dislodged by gentle pipetting 10 times with a P1000 pipette. This method dislodges neurons, while leaving nonneuronal cells attached to the coverslip, so that the replated suspension is very clean. The total suspension of dislodged cells from 1 well was replated onto a new coverslip. The concentration of resuspended cells prior to replating was too low to accurately quantify with a hemacytometer, but similar densities between replated GFP AV– and Cre AV–treated neurons were confirmed after replating and again after fixation and staining. For the rat FL2-knockdown experiments in Supplemental Figure 6, the same dissection and culturing protocol was followed, except DRGs were harvested from 8- to 12-week-old Sprague-Dawley rats (Charles River Laboratories), and cells were transduced with adeno-associated virus 5 containing a control scrambled shRNA plasmid or an FL2 shRNA plasmid (Vector Biolabs). shRNA sequences were subcloned into a D2-U6 vector. Scrambled sequence: CAACAAGATGAAGAGCACCAACTCGAGTTGGTGCTCTTCATCTTGTTGTTTTTG; FL2 sequence: CACCGCTGGAGCCCTTTGACAAGTTCTCGAGAACTTGTCAAAGGGCTCCAGCTTTT.

### Immunocytochemistry.

Neurons were rinsed in prewarmed HBSS and then fixed in warm 4% paraformaldehyde (PFA), 0.15% glutaraldehyde, and 0.1% Triton X-100, in BRB80 (80 mM PIPES, 1 mM EGTA, 1 mM MgCl_2_, pH 6.9) at 37°C for 10 minutes and then room temperature (RT) for 10 more minutes. Cells were rinsed twice in PBS, incubated with 5–10 mg/mL sodium borohydride in PBS for 15 minutes, rinsed, and then permeabilized with 0.4% Triton X-100 in PBS for 5 minutes. Cells were rinsed with PBS 3 times and incubated in blocking buffer (5% normal goat serum, 0.2% sodium azide, 0.1% Triton X-100 in PBS) for 1 hour at RT or overnight at 4°C. Primary antibodies diluted in blocking buffer were applied for 2 hours at RT. After three 5-minute washes in PBS + 0.05% Tween, secondary antibodies were applied for 1 hour at RT; then coverslips were washed and mounted. Primary antibodies used: rabbit anti-TUBB3 (BioLegend; catalog 802001), anti-mouse tyrosinated tubulin (MilliporeSigma; T5168), anti-mouse acetylated tubulin (Abcam; ab24610). Secondary antibodies used: Cy5–anti-rabbit (Jackson ImmunoResearch; 111-175-144), Alexa Fluor 568–anti-mouse (Invitrogen, Thermo Fisher Scientific; A11004). Actin was stained with phalloidin–Alexa Fluor 488 (Invitrogen, Thermo Fisher Scientific; A12379).

### Imaging.

Neurons were imaged using an EVOS FL Auto epifluorescence microscope (Life Technologies, Thermo Fisher Scientific) at ×10 original magnification for neurite length analysis and aggrecan stripe assay analysis and ×40 original magnification for quantitative immunofluorescence and GC morphology experiments.

### Morphometric analyses.

For neurite length and branching analysis, the semiautomated ImageJ plug-in NeuronJ was used (NIH). The length of the longest neurite was measured to quantify average axon length. All neurite-bearing neurons that had a primary neurite that was greater than the diameter of the soma, and that was clearly discernible and traceable (i.e., not considerably overlapping with other neurites), were included in the analysis. A smaller subset of neurons (the first 50–60 neurons per experiment) was used to trace all neurites for quantification of the sum length of neurite outgrowth and average numbers of primary, secondary, and tertiary neurites. All neurites more than 10 μm in length were included in this analysis. Neurons that had neurites that overlapped with neurites from other neurons were excluded from analysis. For GC morphology, any thin actin protrusion greater than 0.5 μm in length was considered a filopodia. To measure MT splay in the GC, the distance between the 2 most peripheral MT plus ends in the GC was measured.

### Quantitative immunofluorescence analysis.

To quantify the ratio of tyrosinated/TUBB3, the 2 fluorescence channels were combined into a merged RGB image in ImageJ, and a line was drawn over the distal neurite starting from the GC tip and moving toward the soma. RGB profiler was then used to obtain the intensity values of the 2 channels over each pixel along the line. These values were background corrected and then the ratio calculated along the line. To calculate the ratios of acetylated/ TUBB3, regions of interest (ROIs) were created around the distalmost 50 μM of neurites as well as the proximal-mid sections of neurites in ImageJ, and the total fluorescence in the separate channels was measured for each ROI and background corrected, and then the ratio was calculated. Neurites from a minimum of 28 neurons were analyzed per treatment group per experiment. Experiments were performed in triplicate.

### Aggrecan stripe assay.

Stripes were prepared as previously described (39) with minor modifications. Briefly, aggrecan and Alexa Fluor 568–anti-mouse antibody were mixed in sterile PBS to final concentrations of 0.2 mg/mL aggrecan and 50 μg/mL antibody. A Hamilton syringe was used to inject the mixture into zigzag stripe silicon matrices (purchased from Martin Bastmeyer, Karlsruhe Institute of Technology, Karlsruhe, Germany). The injected matrices were incubated at 37°C for 40 minutes; then 300 μL of PBS was washed through the matrices 3 times, after which the silicon matrices were removed from the dish. The stripe area was then incubated with PDL/LAM solution at 37°C for 60 minutes. The stripe areas were washed with PBS and then culture media applied to the area until immediately before neurons were seeded onto stripes. The presence of CSPG on the stripes was confirmed by staining with an anti-CSPG antibody (MilliporeSigma, catalog C8035), 3 days after incubation with media. For the experiments, AV-treated neurons were fixed and immunostained for βIII tubulin. Stripes and neurons were imaged at ×10 original magnification on an EVOS FL Auto microscope (Life Technologies, Thermo Fisher Scientific). For quantification of neurite crossings, the following criteria were used: any time an axon’s trajectory came within several micrometers of stripe and then angled away from the stripe to stay within the growth-permissive area, this was counted as a GC turning event. If it was unclear whether a neurite responded to a stripe or not (i.e., if the neurite grazed the side of a stripe boundary, crossing into it briefly but continuing on its trajectory back into the permissive stripe zone), this neurite was not included in the total neurite count.

### Sema3A-induced GC collapse analysis.

Neurons were replated as described above. Two days after replating, media were changed to media with 100 ng/mL recombinant mouse Sema3A-Fc (R&D Systems, Bio-Techne) or Fc (R&D Systems, Bio-Techne) as a control for 2 hours, then fixed and immunostained for βIII tubulin and actin. GCs were imaged at ×40 original magnification. For GCs to be counted as collapsed, GC MTs had to be bundled and no could be filopodia present (the width of these collapsed GCs was less than 2 μm). Over 100 neurites from 28–32 neurons were analyzed per condition each experiment. Experiment was performed in quadruplicate for AV-treated neurons and in triplicate for non–AV-treated control neurons. All imaging and analysis for this experiment were performed in a blinded manner.

### siRNA nanoparticle synthesis.

Nanoparticles were prepared as previously described (22) with minor modifications: 500 μL of tetramethyl orthosilicate (TMOS) was hydrolyzed in the presence of 100 μL of 1 mM HCl by sonication on ice for about 15 minutes, until a single phase formed. The hydrolyzed TMOS (100 μL) was added to 900 μL of 10 μM of pooled siRNA against rat FL2 (siRNA from MilliporeSigma: SASI_Rn02 00314854, target sequence: CTGGATGTCTCCTCCACCA; SASI_Rn02 00314855, target sequence: CAGAGGATGGGACCGGCAA; SASI_Rn02_ 00389576, target sequence: CCTCCAACCTCCTCAAGAG) or the negative control siRNA (MilliporeSigma, Universal Negative control B) solution containing 10 mM phosphate, pH 7.4. A gel was formed within 10 minutes. The gel was frozen at −80°C for 15 minutes and lyophilized. The dried sample was ground into a fine powder with a mortar and pestle. The nanoparticles were then resuspended in sterile PBS at an siRNA concentration of 10 μM and stored at –80°C until immediately before use.

### CN injuries and treatments.

Animals were anesthetized with ketamine and xylazine. A midline abdominal incision was made and the CN exposed and isolated. Three rat models of CN injury were used: mild (a smooth clamp applied for 2 minutes to the CN), moderate (a serrated clamp applied for 4 minutes to the CN), and severe (CN transection). In the case of the crush injury studies, 10 μL of a 10 μM control or FL2-npsi suspension was applied directly onto the nerve injury site. For the transection model, a collagen chondroitin sulfate–hardened microgel (wafer) containing control or FL2-siRNA (synthesized by and purchased from BioADD Laboratory at Stanford University) was applied directly to the wound. The approximate concentration of siRNA in the wafer was 13.3 μg siRNA/100 mg of wafer, and approximately 15 mg of wafer was applied. The wafer completely covered the nerve injury site as well as the major pelvic ganglion.

### Cavernosometry.

Animals were anesthetized with a Nembutal Sodium solution (Oak Pharmaceuticals) 50 mg/mL, and erectile function was determined by electrostimulation of the CNs and by measuring ICP: through a repeat midline abdominal incision, the CNs were exposed and isolated. Cannulas were inserted into the crura at the base of the penis and the carotid artery to measure the ICP and BP, and a bipolar stainless steel electrode, inserted above the site of nerve transection, was used to directly stimulate the CN (probes 2 mm in diameter, separated by 1 mm) via a signal generator and a custom-built constant-current amplifier generating monophasic rectangular pulses with stimulus parameters ranging from 1.5–6 mA, 20 Hz, pulse width of 0.2 ms, and duration of 50 s. The ICP and BP were recorded in all rats using a bioinformation acquisition system. The maximal ICP/BP ratios in the experimental animals were calculated for each level of stimulation.

### Quantification of FL2 mRNA expression by RT-qPCR.

To analyze FL2 mRNA expression in vitro, RNA was isolated from cell cultures using TRIzol (Thermo Fisher Scientific) following manufacturer’s protocol. A total of 200–300 ng of RNA was reverse-transcribed using the SSVilo IV kit (Invitrogen, Thermo Fisher Scientific). For adult DRG cultures, neurons from 4 mice were pulled into 1 RNA extract for each treatment group. B35 cells (purchased from ATCC) were serum starved for 24 hours prior to treatment with npsi and then harvested 24 hours after Npsi treatment. To verify downregulation of FL2 mRNA following wafer treatment in vivo, rat MPG were harvested and kept in RNAlater (QIAGEN) immediately after cavernosometry. MPG were homogenized using a bullet blender (Next Advance), and RNA was reverse-transcribed using High-Capacity cDNA Reverse Transcription Kit (Ambion) (500 ng/reaction). *Power* SYBR Green Master Mix was used for qPCR, using the 7300 Real-Time PCR system (Applied Biosystems, Thermo Fisher Scientific). The following primer sequences were used: mouse Fignl2: GTTCACACTCCTCACACCTG and GCTCCTAGATCCCTTCATGTTC; rat Fignl2: GAGTTGCTGCAGTGTGAATG and CTCTGTGCTTCTGTCTCTGT; β-actin (mouse and rat): CGTTGACATCCGTAAAGACC and TCTCCTTCTGCATCCTGTCA; tdTomato: CACGCTGATTCTACAAGGTGAA and CCCATGGTCTTCTTCTGCATTA. RPL19 was used as reference gene for the in vivo experiments (ACCCCAATGAAACCAACGAA and TCAGGCCATCTTTGATCAGCTT). Results were analyzed using the comparative 2^-ΔΔCt^ method.

### TEM analysis.

Distal nerve segments from 1 (unstimulated) regenerated FL2 siRNA wafer–treated nerve and 1 uninjured control animal were harvested while the animal was anesthetized with ketamine/xylazine. The samples were fixed with 2% PFA and 2.5% glutaraldehyde in 0.1 M sodium cacodylate buffer, postfixed with 1% osmium tetroxide followed by 2% uranyl acetate, dehydrated through a graded series of ethanol, and embedded in LX112 resin (LADD Research Industries). Ultrathin (80 nm) sections were cut on a Leica EM Ultracut UC7, stained with uranyl acetate followed by lead citrate, and viewed on a JEOL 1200EX transmission electron microscope at 80 kV.

### Immunohistochemistry.

MPG were harvested after sacrifice (by CO_2_) and fixed for 20 hours in 4%PFA, transferred to 30% sucrose for 48 hours, and then cryosectioned in 10 μm slices longitudinally. Sections were collected sequentially onto slides. Per MPG, 2–3 sections, 100–150 μm apart from each other, were used for quantification of nNOS^+^ somas. Sections were immunostained for nNOS (Santa Cruz Biotechnology; SC-5302) and tyrosine hydroxylase (Abcam; ab112) (as a counterstain to visualize sympathetic neurons) as well as DAPI according to standard IHC protocol. Slides were imaged on an EVOS FL Auto epifluorescence microscope (Life Technologies, Thermo Fisher Scientific) using a ×10 objective. nNOS^+^ somas in the caudal region of the MPG proximal to the CN were quantified (areas ranged 0.25–0.5 mm^2^). If there was a tear in the tissue in the analyzed region, this area was subtracted from the total area. Eight to 10 sections from 3–4 MPG were analyzed. Imaging and analysis for these experiments were performed in a blinded manner.

### Corporal tissue homogenate preparation and Western blot analysis.

A 0.5 cm segment of the mid penile shaft from each rat was flash frozen after sacrifice. The samples were then minced and homogenized in lysis buffer (50 mM Tris, pH 7.5, 2 mM EDTA, 2 mM EGTA, 150 mM NaCl, 1% Triton X-100, and 10% vol./vol. glycerol) using a Bullet Blender (Next Advance). Thirty to 50 μg of protein was loaded into 4%–20% gradient gels for SDS-PAGE at 35 mA using the Bio-Rad Mini-PROTEAN system, then protein transferred onto a nitrocellulose membrane for 1.5 hours at RT at 100 V. Blots were blocked in 5% milk in TBS, then incubated overnight with primary antibodies against nNOS (Santa Cruz Biotechnology; SC-5302) (1:1000) or GAPDH (Fitzgerald; 10-1500) (1:20,000) and incubated with secondary antibodies conjugated to horseradish peroxidase for 1 hour at RT. Blots were visualized using the iBright system, and densitometry was performed using iBright software automated analysis.

### Statistics.

GraphPad was used to run statistical analyses for all experiments. Unpaired 2-tailed Student’s *t* test or 1-way ANOVA with Tukey’s test was used for cavernosometry data analysis, and for cell biology analyses, Welch’s *t* test was used. *P* < 0.05 was considered significant. Unpaired 2-tailed Student’s *t* test was used to analyze qPCR data. For Western blot and histological analyses comparing 3 groups, 1-way ANOVA with either Tukey’s or Dunnett’s T3 post hoc tests was used.

### Study approval.

Animal experiments were performed according to the guidelines published by the Institute of Laboratory Animal Resources of the National Research Council, and animal care for this study was approved by the Institutional Animal Care and Use Committee of the Albert Einstein College of Medicine.

## Author contributions

LB, KPD, and DJS wrote the manuscript; LB designed and performed cell biology experiments, histology, Western blot experiments, and analyses; MT performed all surgeries, cavernosometry, and ICP/BP analysis; AHK assisted with morphometric analysis of neurons and maintenance and breeding of the FL2 conditional KO mouse colony; GAV performed qPCR of MPG tissue samples; RAC contributed intellectually and executed early pilot studies using siRNA in rat neurons; OV assisted with morphometric analysis of rat neurons; PN and JF synthesized nanoparticles; and KPD and DJS conceived of the project.

## Supplementary Material

Supplemental data

## Version 1 04/19/2021

In-Press Preview

## Version 2 05/10/2021

Electronic publication
