# Supplementary material for: Fidgetin-like 2 negatively regulates axonal growth and can be targeted to promote functional nerve regeneration
Source: JCI Insight. 2021 May 10;6(9):e138484. doi: 10.1172/jci.insight.138484 (PMC8262307; doi:10.1172/jci.insight.138484)
Supplement: Supplemental data [file jciinsight-6-138484-s076.pdf]

Supplemental Information:

Figure S1. Insertion of loxP sites and IRES-tdTomato gene at FL2 locus.
Figure S2. Flp-mediated excision of neomycin cassette.
Figure S3. Representative Southern blot results of the animals of interest.
Figure S4. PCR identification of the FL2 neo-deleted knock-in allele.
Figure S5. PCR identification of the Fignl-2 knock-out allele.
Figure S6. Knockdown of FL2 in rat DRGs promotes axonal regeneration.
Figure S7. Correlation of neurite branch points and length of longest neurite.
Figure S8. FL2 knockout does not significantly alter growth cone morphology.
Figure S9. Efficacy of nanoparticle-encapsulated siRNA against FL2 confirmed in vitro.

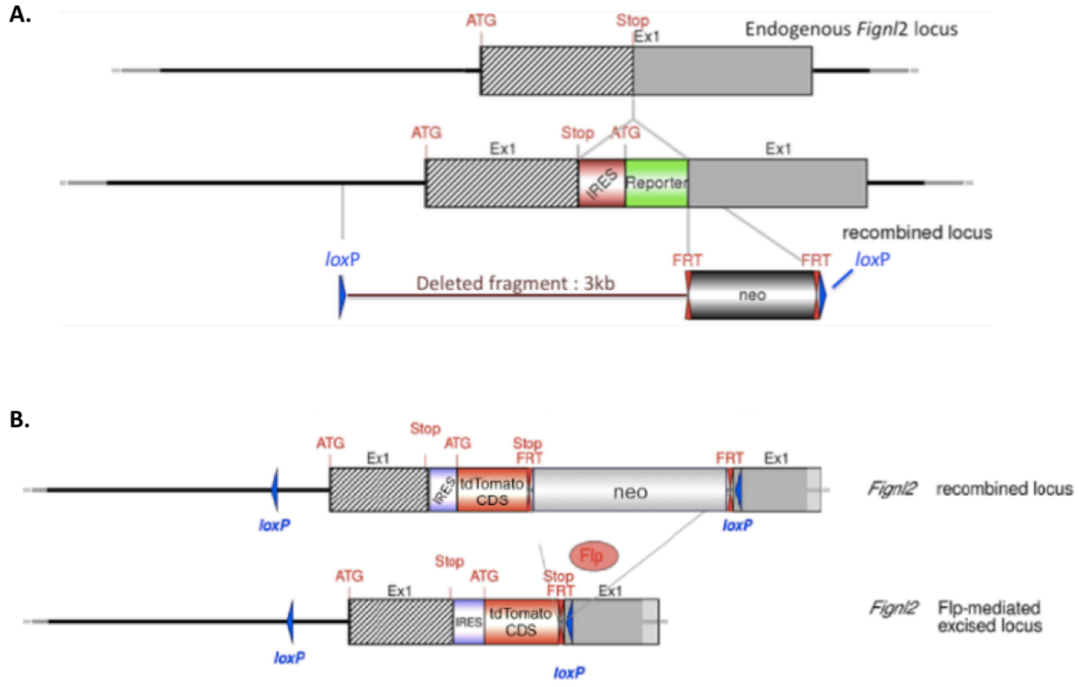

Figure S1: Insertion of loxP sites and IRES-tdTomato gene at FL2 locus. A) Schematic representation of the selected targeting strategy. Hatched rectangles represent Fignl-2 coding sequences, grey rectangles indicate non-coding exon portions and solid lines represent chromosome sequences. The neomycine positive selection cassette is indicated. loxP sites are represented by blue triangles and FRT sites by double red triangles. The initiation (ATG) and Stop (Stop) codons are indicated. The size of the flanked Fignl-2 sequence to be deleted is specified (3kb). **B)** Scheme of Flp-excision at the *Fignl2* recombined locus. Diagrams not depicted to scale.

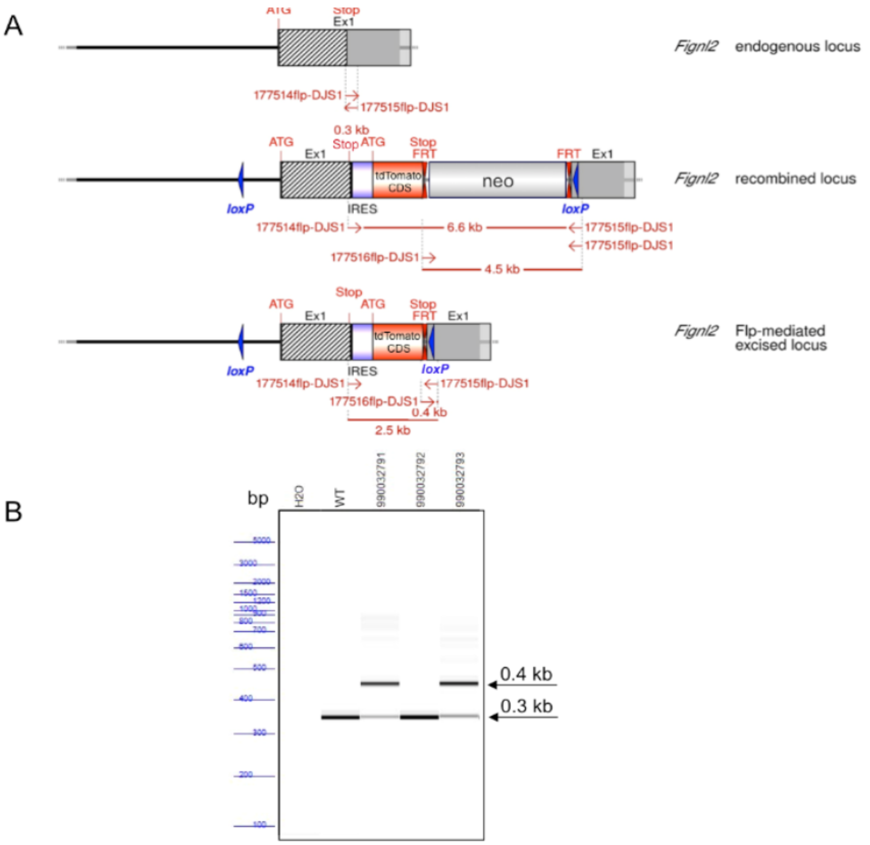

Figure S2: Flp-mediated excision of neomycin cassette. (A) Schematic representation of the Fignl2 wild-type, non-excised recombined and conditional Knockout alleles with the binding sites of the screening primers. (B) PCR using C57BL/6 wild-type genomic DNA (WT) was used as positive control. PCR without DNA as template (H2O) served as a negative control. PCR fragments were separated by capillary electrophoresis using AATI ZAGTM Fragment Analyzer and were analyzed using PROSize 2.0 analytical software. Note: Due to the large size of the 2.5 kb Flp-mediated excised allele PCR product, its amplification did not occur.

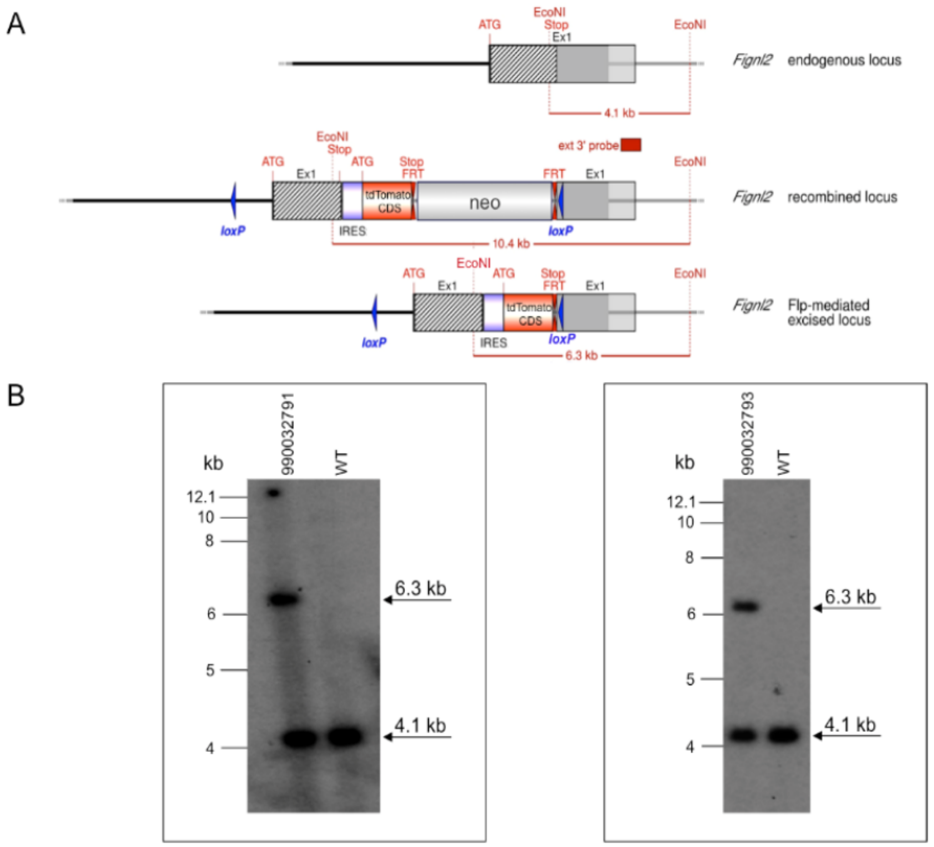

Figure S3: Representative Southern blot results of the animals of interest. (A) Schematic representation of the *Fignl2* wild-type, non-excised recombined and conditional Knockout alleles. (B) The genomic DNA of the tested animals was compared to C57BL/6 wild-type genomic DNA (WT).

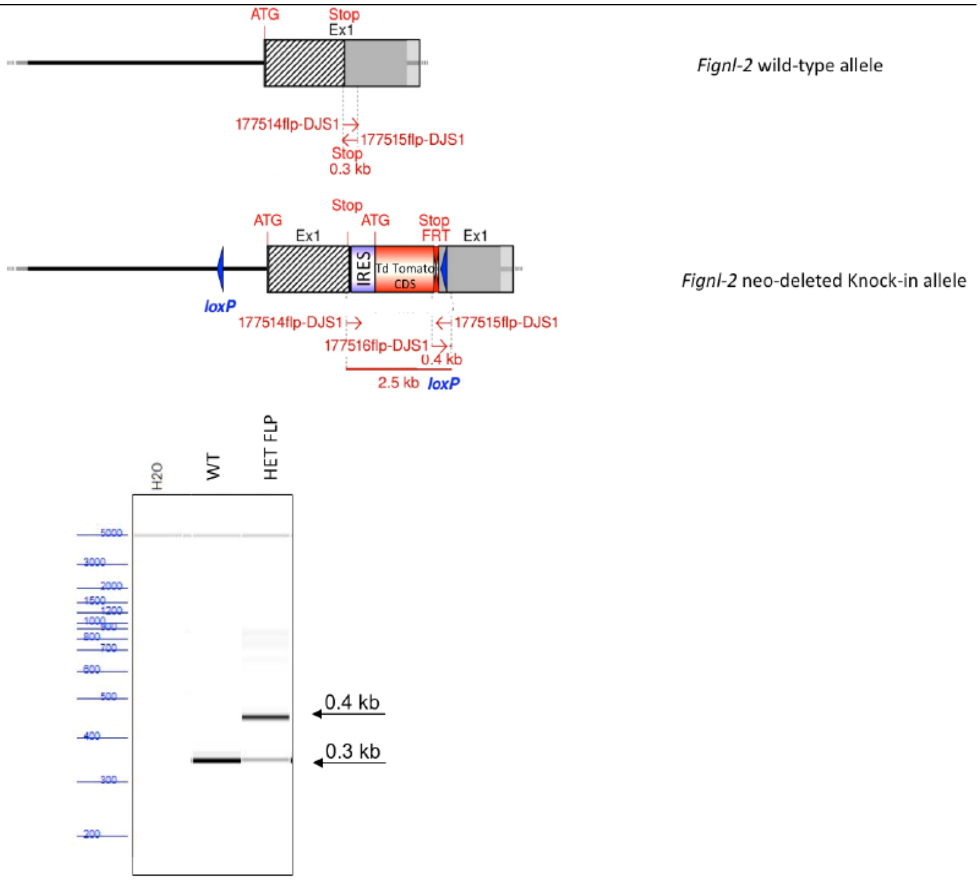

Figure S4: PCR identification of the FL2 neo-deleted knock-in allele. A) Schematic representation of the Fignl-2 wild-type and neo-deleted Knock-in alleles with the binding sites of the screening primers. B) The optimized PCR screening was conducted using genomic DNA of heterozygous conditional/ knock-out animals (HET FLP). PCR with C57BL/6 wild-type genomic DNA (WT) and without DNA (H2O) were used as positive and negative controls, respectively. PCR picture was obtained after loading the PCR reactions on the LabChip® system from Caliper LifeSciences.

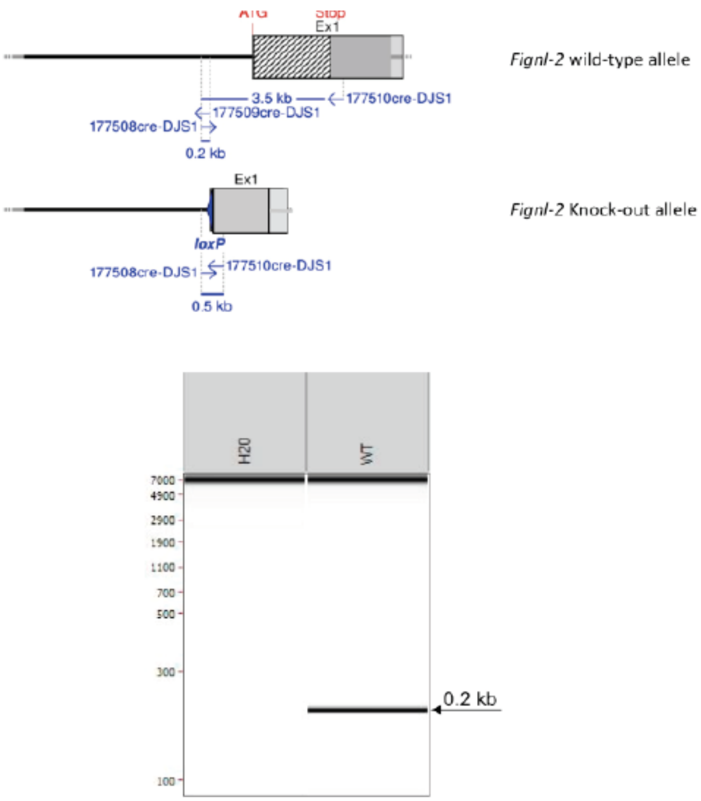

Figure S5: PCR identification of the Fignl-2 Knock-out allele. A) Schematic representation of the *Fignl-2* wild-type and Knock-out alleles with the binding sites of the screening primers. B) The optimised PCR screening was conducted using C57BL/6 wild-type genomic. PCR without DNA (H2O) was used as negative control. PCR picture was obtained after loading the PCR reactions on the LabChip® system from Caliper LifeSciences.

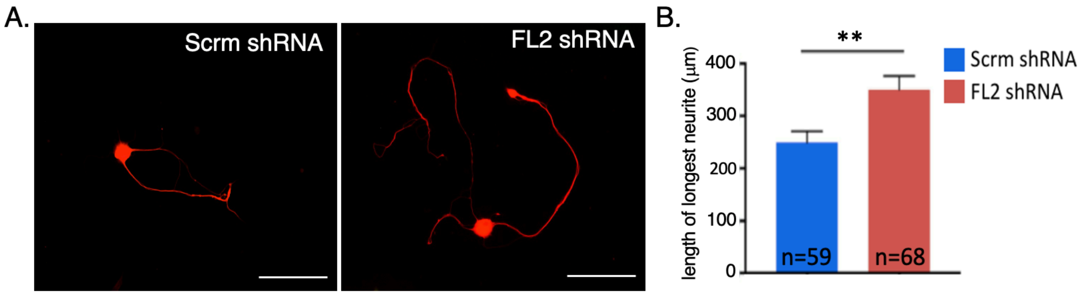

Figure S6. Knockdown of FL2 in rat DRGs promotes axonal regeneration. Preliminary study with adult rat dissociated DRG neurons, replated 5 days after transduction AAV5 containing with control (scrambled) shRNA or FL2 shRNA plasmids, and fixed 72 hours post replating, immunostained for microtubules (TUBB3). FL2 shRNA expressing neurons had significantly longer axons than control treated neurons. **A)** Immuno-micrographs of replated neurons expressing Scrm shRNA or FL2 shRNA, 72 hours after replating. Scale bar = 100 μm. **B)** Graph of the mean length of longest neurites of Scrm shRNA and FL2 shRNA expressing cells (control shRNA n = 59, FL2 shRNA n = 68, student's t-test, $p < 0.01$).

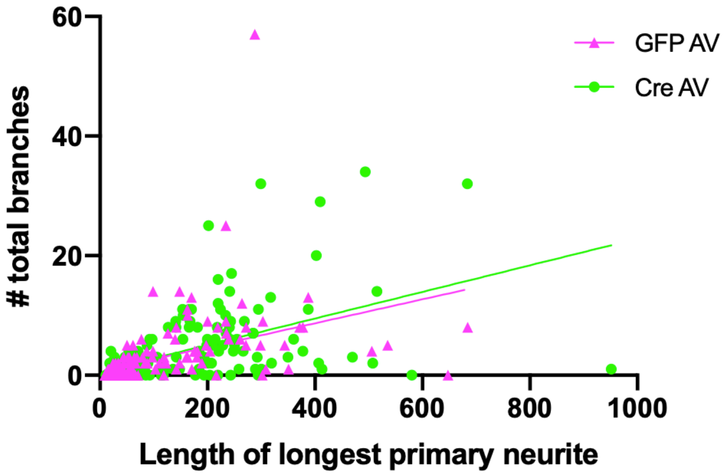

Figure S7. Number of branch points plotted against length of longest neurite. Total number of branch points (the sum of secondary and tertiary neurites) plotted against the length of the longest primary neurite in GFP and Cre AV treated neurons.

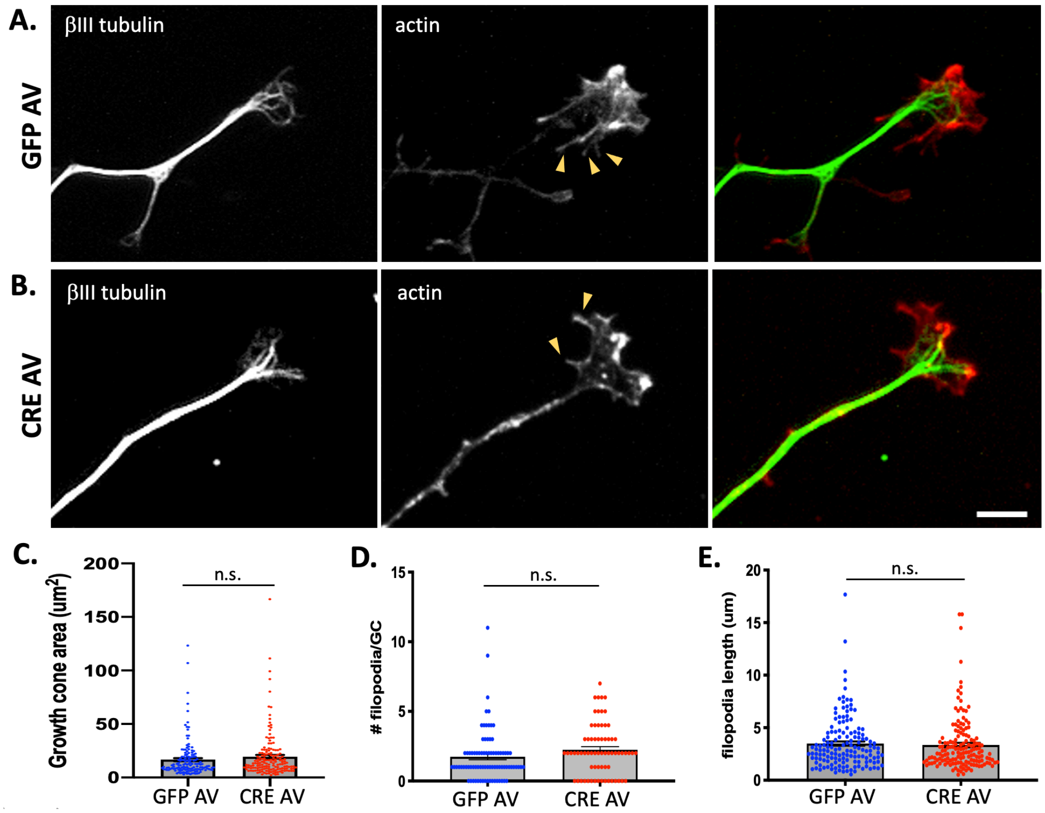

Figure S8. FL2 knockout did not significantly impact growth cone size, filopodia number, of filopodia length in replated adult DRG neurons. A, B) Immuno-micrographs of two dynamic growth cones of GFP AV (A) and Cre AV (B)-treated neurons stained for βIII tubulin and actin. Yellow arrowheads point to filopodia (filopodia defined as thin actin-rich projections 0.5 μm or longer extending from the GC). Brightness and contrast adjusted to more clearly depict filopodia and microtubules in the growth cone. Scale bar = 5 μm. **C)** Dot plot of growth cone area measurements for GFP and Cre AV treated and replated neurons 48 hrs post replate (GFP AV: 16.67 ± 1.42, n=147; Cre AV: 19.35 ± 1.66, n=161, p = 0.22, Welch's t-test). **D)** Dot plot of the number of filopodia per growth cone in GFP and Cre AV treated neurons (GFP AV: 1.7 ± 0.20, n= 84; Cre AV: 2.2 ± 0.22, n = 69. p = 0.091, Welch's t-test). **E)** Lengths of filopodia on growth cones of GFP and Cre AV-treated neurons (GFP AV: 3.49 ± 0.20 μm, n=147; Cre AV: 3.35 ± 0.20 μm, n=155, p=0.63, Welch's t-test).

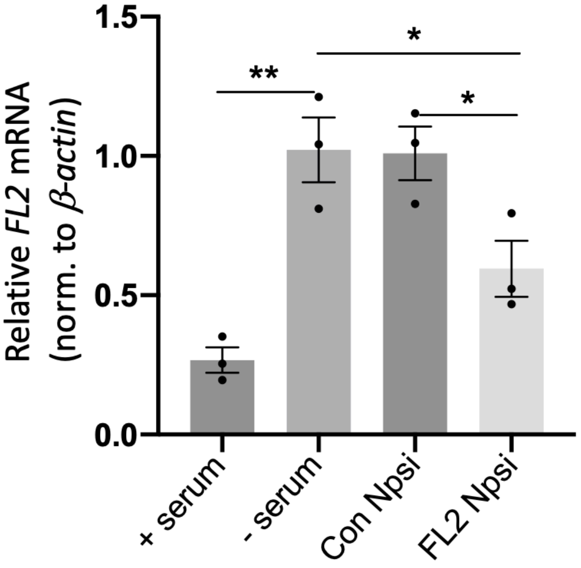

Figure S9. Nanoparticle-encapsulated siRNA (Npsi) effective in eliciting knockdown of FL2 in B35 cells. Relative quantity (RQ) of FL2 mRNA in B35 cells with Npsi treatment, normalized to β-actin. B35 cells are rat neuroblastoma cells that can be induced to develop neurite-like processes by serum starvation. Serum starvation also induces a several-fold increase in the expression of FL2. Cells treated with 20 nM Npsi 24 hours post serum starvation had significantly reduced levels of FL2 mRNA 24 hours post treatment (plus serum: 0.27 ± 0.05; No serum: 1.02 ± 0.12; Control Npsi: 1.01 ± 0.10; FL2 Npsi: 0.59 ± 0.10; Mean ± SEM, **p<0.01; *p<0.05). Experiment performed in triplicate.
